# A Case of Acute Pericarditis With Moderate Pericardial Effusion in a Medically Complex, Bedbound Elderly Patient

**DOI:** 10.7759/cureus.105459

**Published:** 2026-03-18

**Authors:** Garrett A Perchetti, Bilquees Fatima, Roxana Lazarescu

**Affiliations:** 1 Medicine, Wyckoff Heights Medical Center, New York, USA

**Keywords:** acute pericardial effusion, acute pericarditis, anticoagulation, colchicine treatment, pericardial injury, pericarditis

## Abstract

Pericarditis is an inflammatory condition of the pericardium that often presents with chest pain and is diagnosed based on characteristic clinical, electrocardiographic, and imaging findings. We present the case of a 74-year-old female patient with significant comorbidities, including morbid obesity, functional quadriplegia, chronic venous stasis, chronic deep vein thrombosis on anticoagulation, and recent cholecystostomy tube placement, who developed acute pericarditis with a moderate pericardial effusion. Despite initial electrocardiographic findings concerning for acute coronary syndrome, serial cardiac biomarkers were negative, and imaging confirmed pericardial inflammation without tamponade physiology. The patient was successfully managed with non-steroidal anti-inflammatory drugs and colchicine, with improvement in symptoms. This case highlights the diagnostic challenges of pericarditis in medically complex patients and emphasizes contemporary guideline-directed management, risk stratification, and multidisciplinary care coordination.

## Introduction

Pericarditis accounts for approximately 5% of emergency department presentations for chest pain unrelated to acute coronary syndrome (ACS) [1]. While its diagnosis and management are well-characterized in the general population, medically complex patients, particularly those who are elderly, bedbound, and burdened with multiple comorbidities, represent a distinctly underrepresented and challenging subgroup in the existing literature. In such patients, atypical presentations, competing diagnoses, and contraindications to standard therapies can obscure the diagnostic picture and complicate management at every step.

Diagnosis requires at least two of the following criteria: characteristic pleuritic chest pain, pericardial friction rub, typical electrocardiographic changes (diffuse ST-segment elevation or PR depression), and new or worsening pericardial effusion [2]. Elevated inflammatory markers such as C-reactive protein (CRP) and erythrocyte sedimentation rate (ESR) further support the diagnosis. In developed countries, most cases are idiopathic or presumed viral; however, secondary etiologies, including autoimmune disease, malignancy, bacterial infection, metabolic disorders, and post-procedural complications, must be systematically excluded [2,3], a task made considerably more difficult when baseline laboratory and imaging abnormalities reflect pre-existing disease rather than acute pathology.

Risk stratification is especially consequential in complex patients. Moderate-to-large pericardial effusions, anticoagulation use, fever, immunosuppression, or a subacute course all warrant inpatient monitoring given the risk of progression to tamponade, features that may co-exist and interact unpredictably in patients with significant baseline illness [2]. Management has evolved substantially over the past decade: combination therapy with non-steroidal anti-inflammatory drugs (NSAIDs) and colchicine is now first-line to reduce recurrence, corticosteroids are reserved for refractory or specific etiologies due to their association with higher recurrence rates [4-6], and interleukin (IL)-1 inhibitors have emerged as targeted therapy for recurrent or refractory disease [3,7]. However, applying these guideline-directed strategies in patients with contraindications, polypharmacy, and concurrent active infections requires individualized clinical reasoning that is rarely addressed in the literature.

We present a case of acute pericarditis with moderate pericardial effusion in an elderly bedbound woman with morbid obesity, functional quadriplegia, chronic anticoagulation, and concurrent active infections; this constellation of factors complicated diagnosis, risk stratification, and treatment selection at every stage. This case highlights the real-world challenges of applying contemporary guidelines in medically complex patients and underscores the value of multidisciplinary, individualized care.

## Case presentation

A 74-year-old female patient with a past medical history of morbid obesity (BMI 40 kg/m²), bedbound functional quadriplegia, asthma, chronic venous stasis with left lower extremity deep vein thrombosis (DVT) (on dabigatran), severe right hip osteoarthritis, and a percutaneous cholecystostomy tube placed six months prior to presentation for acute cholecystitis, presented to the emergency department with two days of progressive, sharp, left-sided chest pain and shortness of breath. The pain was intermittent, non-exertional, pleuritic in nature, and worsened with coughing. She reported preceding upper respiratory symptoms, including nasal congestion and dry cough. She denied palpitations, syncope, fever, nausea, or vomiting.

**Figure 1:**
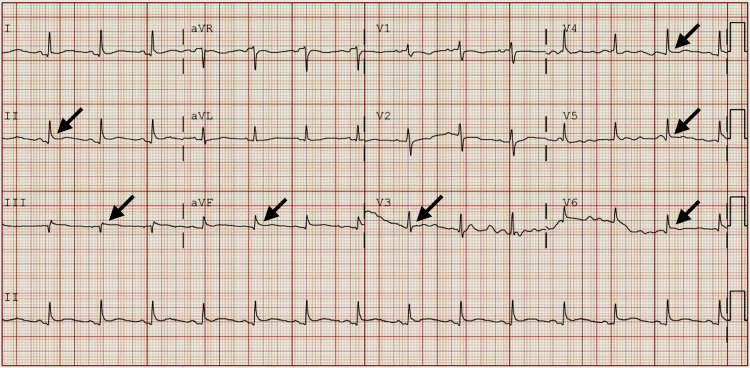
Electrocardiogram done in the emergency department Heart rate: 85 beats per minute, PR:169, QTc: 395, P-axis: 41, QRS-axis: 34, T-axis: 45 Sinus rhythm, low voltage in precordial leads, and ST elevation in leads II, III, AvF, and V3-V6 (arrows)

On admission, the patient was hemodynamically stable (blood pressure 103/60 mmHg, heart rate 89 beats per minute, oxygen saturation 97% on 2L nasal cannula). Electrocardiography demonstrated ST-segment elevation in the inferior and lateral leads (Figure 1), raising concern for possible ST-elevation myocardial infarction. However, serial high-sensitivity troponins remained negative, and there were no regional wall motion abnormalities on imaging.

**Figure 2:**
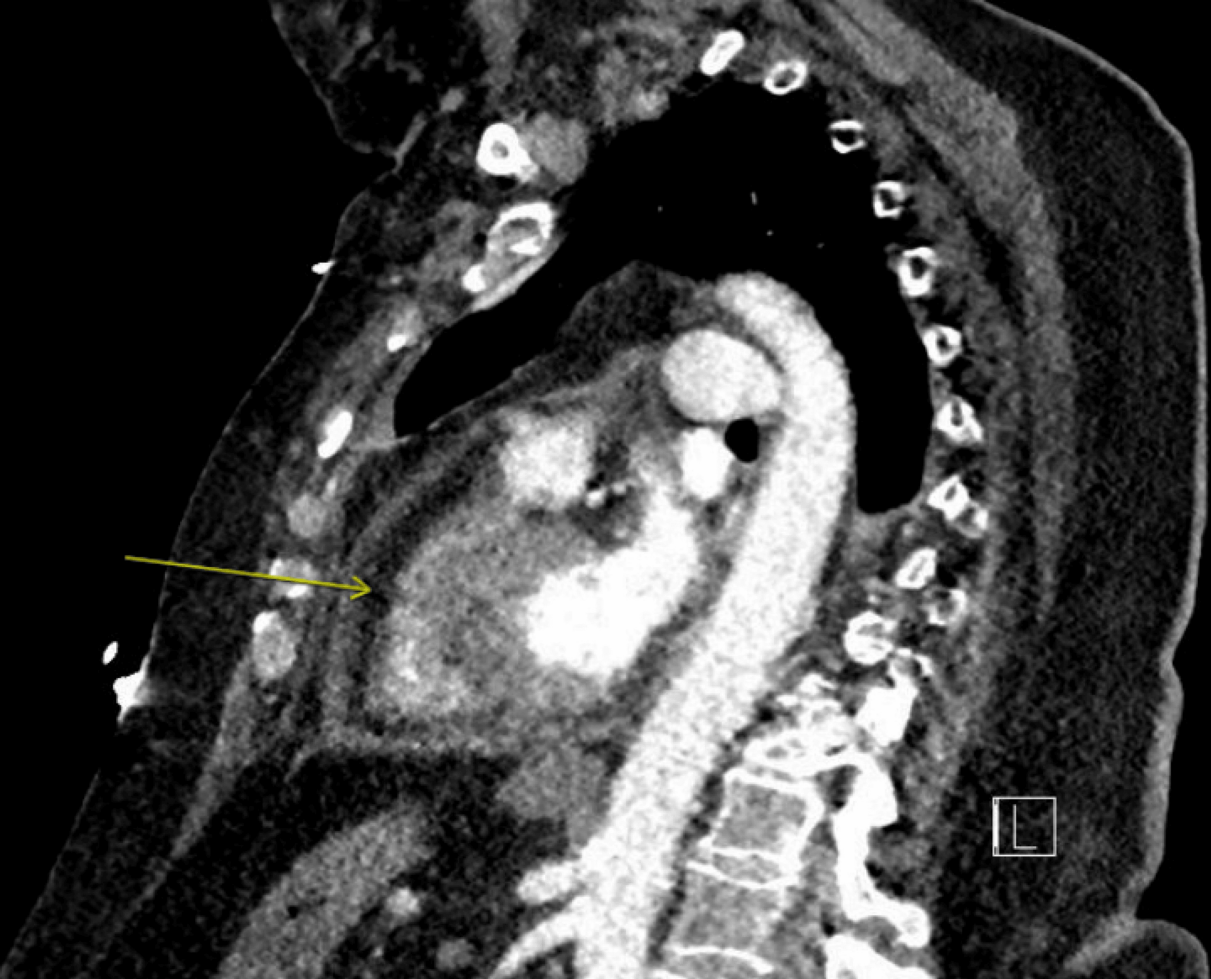
CT of the chest (sagittal cross-section) showing a moderate sized pericardial effusion (arrow)

Chest radiography revealed pulmonary vascular congestion, cardiomegaly, and bibasilar atelectasis. CT angiography of the chest excluded pulmonary embolism and aortic dissection but demonstrated a moderate pericardial effusion measuring approximately 2.2 cm, along with small bilateral pleural effusions and mild cardiomegaly (Figure 2). Laboratory evaluation showed markedly elevated inflammatory markers (CRP 181 mg/L; ESR 98 mm/hour), mildly elevated procalcitonin (0.45 ng/mL), and B-type natriuretic peptide (BNP) 131 pg/mL (Table 1). Autoimmune testing (antinuclear antibodies (ANA), double-stranded DNA (dsDNA), rheumatoid factor) was negative. Respiratory viral panels, including COVID-19, Influenza, respiratory syncytial virus (RSV), and Coxsackie virus, were negative and blood cultures showed no growth (Table 2).

**Table 1:** Laboratory values over hospitalization CRP: C-reactive protein; AST: aspartate aminotransferase; ALT: alanine transaminase; BUN: blood urea nitrogen; BNP: B-type natriuretic peptide

Lab Test	Hospital Day 1	Hospital Day 2	Hospital Day 3	Trend	Reference Range
WBC (K/µL)	11.0 ↑	—	8.1	Improved	4.5–10.9
Neutrophils (%)	85.5 ↑	—	72.1 ↑	Improving	50–70
Lymphocytes (%)	5.9 ↓	—	15.8 ↓	Improving	25–50
Hemoglobin (g/dL)	13.3	—	11.9 ↓	Downtrend	12.2–15.0
CRP (mg/L)	168 ↑	181 ↑	—	Worsened (early)	0.2–3.0
Procalcitonin (ng/mL)	—	0.45 ↑	—	Elevated	0.05–0.09
ESR (mm/hr)	—	98 ↑	—	Elevated	0–30
Total Bilirubin (mg/dL)	2.0 ↑	—	0.8	Resolved	0.2–1.0
Direct Bilirubin (mg/dL)	1.12 ↑	—	—	Elevated	0.10–0.20
Alkaline Phosphatase (U/L)	255 ↑	—	233 ↑	Slight ↓ but high	45–117
AST (U/L)	46 ↑	—	33	Normalized	15–37
ALT (U/L)	36	—	36	Stable	12–78
Albumin (g/dL)	2.8 ↓	—	2.5 ↓	Worsened	3.4–5.0
BUN (mg/dL)	14	—	21 ↑	Increased	7–18
Creatinine (mg/dL)	0.84	—	0.88	Stable	0.55–1.30
Glucose (mg/dL)	152 ↑	—	118 ↑	Improved	74–106
BNP (pg/mL)	131 ↑	—	—	Mildly elevated	0–100
Troponin (ng/L)	14.2	—	—	Normal	3.0–58.9

**Table 2:** Immunologic and serologic studies ANA: antinuclear antibody; dsDNA Ab: anti-double-stranded deoxyribonucleic acid antibodies; C-ANCA: antineutrophil cytoplasmic autoantibody, cytoplasmic; P-ANCA: perinuclear anti-neutrophil cytoplasmic antibody

Test	Result	Reference	Interpretation
Coxsackie Virus Panel	<1:8	<1:8	Negative
ANA	1:40	<1:40 negative	Low-positive
ANA Pattern	Cytoplasmic, Fibrillar (AC-15,16,17)	—	Nonspecific
dsDNA Ab	<1	Negative	Negative
C-ANCA	<1	Negative	Negative
P-ANCA	<1	Negative	Negative
Fungal Culture (Biliary Fluid)	No fungal elements	—	Negative

Transthoracic echocardiography demonstrated preserved left ventricular ejection fraction (55-60%) with a small-to-moderate circumferential pericardial effusion without echocardiographic evidence of tamponade physiology (no right atrial or ventricular diastolic collapse). Given fulfillment of diagnostic criteria for acute pericarditis and absence of tamponade, the patient was treated with colchicine (loading dose 1.2 mg twice, followed by maintenance 0.6 mg twice daily for four weeks) and ibuprofen 600 mg three times daily for two weeks with gastric prophylaxis. Anticoagulation was temporarily held until tamponade was excluded, after which dabigatran was transitioned to apixaban in consideration of bleeding risk.

Additional issues during hospitalization included infection of the cholecystostomy tube with *Pseudomonas aeruginosa,* managed with levofloxacin 750 mg daily for seven days and outpatient interventional radiology and gastroenterology follow-up; bilateral venous stasis dermatitis with superimposed cellulitis, managed with doxycycline and wound care consultation; and chronic DVT, continued on apixaban. The patient’s chest pain improved significantly during hospitalization, inflammatory markers trended downward, and she was discharged in stable condition with cardiology follow-up for repeat echocardiography.

## Discussion

This patient met three of the four diagnostic criteria for acute pericarditis: pleuritic chest pain, characteristic ECG changes, and new pericardial effusion, with supportive elevation in inflammatory markers [2]. Although ST-segment elevations initially raised concern for ACS, negative serial troponins and absence of regional wall motion abnormalities supported pericarditis as the underlying diagnosis.

According to contemporary expert consensus guidance, moderate effusion (>20 mm), anticoagulation use, and significant comorbidities justify inpatient monitoring [2]. The primary concern in such cases is progression to cardiac tamponade, particularly in anticoagulated patients [8,9]. Close observation and echocardiographic evaluation allowed safe continuation of anticoagulation once tamponade was excluded. The transition from dabigatran to apixaban was made in consideration of the potential need for dose flexibility and the slightly more favorable bleeding profile of apixaban in the setting of concurrent soft tissue infection and hemodynamic borderline stability. This decision reflects the broader challenge of anticoagulation management in pericarditis, where continuation is necessary to prevent thromboembolic complications from chronic DVT, yet carries an incremental risk of hemorrhagic effusion expansion if tamponade physiology were to develop [10].

While idiopathic or viral etiologies account for most cases in developed countries [1,2], secondary causes must be systematically ruled out. In this case, Autoimmune disease was excluded with negative ANA, dsDNA, and RF. Bacterial pericarditis was unlikely given the absence of bacteremia, hemodynamic instability, and the lack of purulent pericardial fluid, which argued against a suppurative etiology. The mildly elevated procalcitonin level was attributed to concurrent biliary and soft-tissue infections rather than to pericardial infection per se. Malignancy was not suggested by imaging. Uremic pericarditis was excluded by normal renal function. Post-procedural causes were unlikely, given no recent cardiac instrumentation. Given the preceding respiratory symptoms and markedly elevated CRP, viral pericarditis was the most probable etiology.

Combination therapy with NSAIDs and colchicine is supported by randomized trials and meta-analyses demonstrating a significant reduction in recurrence and symptom persistence [4]. Moreover, a landmark randomized trial in 2013 by Imazio et al. in 2013 demonstrated that colchicine added to conventional therapy reduced incessant or recurrent pericarditis from 37.5% to 16.7% [5]. Corticosteroids were avoided due to their association with increased recurrence risk in idiopathic pericarditis [6]. Recent advances over the past five years have expanded therapeutic options for recurrent or refractory pericarditis. IL-1 inhibitors such as anakinra and rilonacept target the autoinflammatory pathway central to recurrent disease and significantly reduce recurrence rates [3,7]. Although not required in this case, awareness of these therapies is essential in modern practice.

Emerging consensus emphasizes the role of multimodal imaging, including echocardiography, cardiac CT, and cardiac MRI, in refining diagnosis, characterizing effusion size, and evaluating for complications [10,11]. In this case, CT imaging incidentally quantified effusion size and excluded alternative thoracic pathology, while echocardiography assessed hemodynamic impact. Our case features markedly elevated CRP. Typically, normalization and downward trends from elevation have been used as prognostic markers and treatment end-points [12]; however, our patient was lost to follow-up, and no additional CRP measurements were taken after receiving treatment, thus representing a limitation of our study. Notably, CRP continued to rise from 168 to 181 mg/L over the first two days of hospitalization before treatment was fully established, a pattern consistent with the natural early trajectory of acute pericarditis prior to anti-inflammatory therapy taking effect. Serial CRP monitoring following initiation of NSAIDs and colchicine is recommended to guide treatment duration and identify patients at risk for recurrence, and the absence of post-treatment values in this case precluded full application of this prognostic strategy.

## Conclusions

This case illustrates the diagnostic and management challenges of acute pericarditis in a medically complex, bedbound elderly patient. The initial electrocardiographic findings mimicked ACS, underscoring the importance of integrating clinical context, serial biomarkers, and multimodal imaging before arriving at a diagnosis. The patient's multiple comorbidities, including anticoagulation use, morbid obesity, and concurrent infections, complicated risk stratification and required careful, individualized decision-making at each step of her care. Temporary suspension of anticoagulation until tamponade was excluded, followed by a thoughtful transition to an alternative agent, reflected the need for multidisciplinary coordination in such patients. Treatment with ibuprofen and colchicine led to meaningful symptom improvement and a downward trend in inflammatory markers prior to discharge. Outpatient cardiology follow-up with repeat echocardiography was arranged to monitor effusion resolution and assess for recurrence. Taken together, this case demonstrates that timely diagnosis, careful risk stratification, and coordinated inpatient management can achieve favorable outcomes even in patients with significant baseline complexity.
